# A Portable Fluorometer Detects Significantly Elevated Cell-Free DNA in Tracheal Wash and Bronchoalveolar Lavage Fluid in Horses with Severe Asthma

**DOI:** 10.3390/ani15233483

**Published:** 2025-12-03

**Authors:** Bethanie L. Cooper, Kallie J. Hobbs, Rosemary Bayless, Austen Stinson-Miller, Erika Gruber, Kate Hepworth-Warren, Jean-Pierre Lavoie, M. Katie Sheats

**Affiliations:** 1 Department of Clinical Sciences, College of Veterinary Medicine, North Carolina State University, Raleigh, NC 27607, USA; bplewis2@ncsu.edu (B.L.C.); klhepwor@ncsu.edu (K.H.-W.); 2 Department of Clinical Sciences, College of Veterinary Medicine, Texas A&M University, College Station, TX 77840, USA; khobbs05@tamu.edu; 3 Department of Molecular Biomedical Sciences, College of Veterinary Medicine, North Carolina State University, Raleigh, NC 27607, USA; rlbayles@ncsu.edu; 4 College of Agriculture and Life Sciences, North Carolina State University, Raleigh, NC 27607, USA; ahstinso@ncsu.edu; 5 Department of Population Health and Pathobiology, College of Veterinary Medicine, North Carolina State University, Raleigh, NC 27607, USA; ejgruber@ncsu.edu; 6 Département de Sciences Cliniques, Faculté de Médecine Vétérinaire, Université de Montréal, St-Hyacinthe, QC J2S 2M2, Canada; jean-pierre.lavoie@umontreal.ca

**Keywords:** cell-free DNA, asthma, neutrophil extracellular traps, horses

## Abstract

Severe equine asthma (sEA) is characterized by increased lower airway neutrophils that contribute to dysregulated inflammation through the release of cytokines, reactive oxygen species and neutrophil extracellular traps (NETs). NETs are composed of cell-free DNA (cfDNA) intercalated with enzymatic proteins and are known to be increased in the lower airway of asthmatic horses. The objectives of this study were two-fold: 1. Determine if cfDNA can be accurately measured in equine bronchoalveolar lavage fluid (BALF) and tracheal wash (TW) with a Qubit 4 fluorometer. 2. Determine whether Qubit-measured cfDNA in BALF or TW is significantly different in horses with sEA, mild/moderate neutrophilic equine asthma, mastocytic equine asthma and healthy horses. A total of sixty-three horses received a physical examination and clinical score followed by a BAL +/− TW. Cell-free DNA was measured using three methods in unfiltered BAL and TW as well as BAL and TW supernatant. Cell-free DNA concentrations were highly correlated between the Qubit 4 fluorometer and NanoDrop spectrophotometer as well as between the Qubit 4 fluorometer and SYTOX green plate-based assay. Cell-free DNA concentrations were highly correlated between unfiltered TW and TW supernatant as well as between unfiltered BALF and BAL supernatant. Cell-free DNA concentrations in BAL and TW supernatant were significantly higher in horses with sEA compared to healthy horses or horses with mild/moderate equine asthma. Cell-free DNA is a biomarker of sEA that can be easily measured in the field with the small portable Qubit 4 fluorometer in BAL and TW fluid. These findings support further investigation of NETs as a biomarker and potential therapeutic target for severe equine asthma.

## Simple Summary

Equine asthma (EA) is an inflammatory disease that causes increased airway mucus, cough and bronchoconstriction. Diagnosis of EA relies on sending airway samples to a lab for analysis; more rapid diagnosis could be achieved with the identification of biomarkers that could be measured immediately on the farm. This study investigated cell-free DNA (cfDNA) as a biomarker of equine asthma. Cell-free DNA is known to be increased in airway samples from humans with asthma because of increased release of neutrophil extracellular traps (NETs). This study measured cfDNA in airway samples from horses with equine asthma and healthy horses using a small, portable, economical device that has potential for stall-side use. Cell-free DNA was measured in airway samples and found to be significantly higher in horses with severe equine asthma compared to mild/moderate asthma and healthy horses. These findings support further investigation of NETs as a biomarker of equine asthma.

## 1. Introduction

Equine asthma is a significant health and welfare concern for horses, whether they are top performance athletes or backyard pleasure animals. Mild/moderate equine asthma (mEA) reportedly affects 60–100% of some equine populations, including racehorses, while severe equine asthma (sEA) reportedly affects 10–20% of adult horses in the northern hemisphere [1,2,3,4,5]. sEA is a chronic, inflammatory lower airway disease characterized by cough, increased respiratory rate and effort at rest, mucus accumulation, bronchial hyperreactivity and elevated lower airway neutrophils [4,5]. Increasing neutrophil percentages in bronchoalveolar lavage fluid (BALF) cytology are highly correlated with asthma severity and decreased lung function [6].

During disease exacerbation, increased lower airway neutrophils contribute to ongoing inflammation and host tissue damage through the release of proteolytic enzymes, reactive oxygen species and neutrophil extracellular traps (NETs) [7,8,9,10]. NETs are extruded strands of neutrophil DNA intercalated with histones and enzymatic proteins. During host defense, neutrophils release NETs to help trap and degrade invading organisms such as bacteria. However, when neutrophil responses become dysregulated, as in equine asthma, over-activated neutrophils release excess NETs, resulting in host tissue damage [11]. Given the role of NETs in inflammation and tissue damage, cell-free DNA (cfDNA), a component of NETs, may present a potential diagnostic biomarker in BALF and therapeutic target for neutrophilic equine asthma. Cell-free DNA is a highly stable molecule that can be easily and economically measured with the Qubit 4 fluorometer and 1X dsDNA high sensitivity assay kit [12,13]. The Qubit 4 fluorometer is small (5″ × 10″), portable and economical with a sample processing and read time of less than three minutes, making it a promising tool for stall-side use.

Current diagnostic approaches for equine asthma include clinical scoring, endoscopy, BALF cytology, tracheal wash (TW) cytology and pulmonary function testing [14]. These diagnostic approaches require specialized equipment and training. The Qubit 4 fluorometer (Thermo Fisher Scientific, Waltham, MA, USA) is a small, portable, economical device that could be used to measure cfDNA in BALF on the farm, negating the need for laboratory submission and delayed diagnosis. cfDNA has emerged as a promising biomarker with diagnostic and prognostic applications. In human medicine, cfDNA is widely studied as a biomarker in sepsis, oncology and respiratory conditions (asthma and chronic obstructive pulmonary disease) [15,16,17]. In equine medicine, cfDNA has been investigated in several inflammatory equine diseases included sepsis and systemic inflammatory response syndrome, uveitis and gastrointestinal disease [18,19,20]. These findings underscore the biological relevance of cfDNA as an indicator of inflammation and tissue injury.

We hypothesized that cfDNA, a component of NETs, could be accurately and feasibly measured in respiratory samples and would be a diagnostic biomarker for neutrophilic endotypes of EA.

## 2. Materials and Methods

All samples were collected between March 2022 and January 2025.

### 2.1. Horses

All procedures were approved by the North Carolina State University Institutional Animal Care and Use Committee (IACUC # 22-262). Thirty-three BALF supernatant samples were obtained from the Université de Montréal Equine Respiratory Tissue Biobank. Thirty additional paired samples (TW and BALF) were collected from university-owned research horses and client-owned horses presenting to the NCSU Equine and Food Animal Veterinary Center for evaluation of respiratory clinical signs. All horses received a physical examination and 23-point clinical score [21]. Horses were excluded from the study if they had a positive tracheal wash culture or had received corticosteroids or bronchodilators within the previous 14 days. Horses were considered healthy if they had normal BALF cytology (<5% neutrophils, <1% eosinophils and <2% mast cells), had a clinical respiratory score < 5 and had no history of asthma. Horses were considered severely asthmatic if they had increased neutrophils on BAL cytology (>20% neutrophils) and clinical respiratory scores ≥ 15 at rest. Horses were considered to have mild/moderate asthma if they had a presenting complaint of cough or altered performance and abnormal BAL cytology (mastocytic: >2% mast cells; neutrophilic: 6–19%) +/− clinical respiratory scores 5–14.

### 2.2. Clinical Scoring

Horses were assigned a 23-point weighted clinical score at the time of respiratory sampling. The previously established 23-point clinical score evaluates respiratory rate, nasal discharge, nasal flaring, abdominal lift, tracheal sounds, bronchial tones, presence of crackles, wheezes and cough [21].

### 2.3. Tracheal Wash Sample Collection and Processing

Horses were sedated with detomidine (0.005–0.01 mg/kg IV) and butorphanol (0.02–0.04 mg/kg IV). For horses receiving both a BAL and TW, the TW was performed first endoscopically. TW fluid was collected as previously described using a 220 cm long, 11 mm diameter video endoscope [22]. The endoscope was advanced to the level of the mid-cervical trachea, and 40 mL of sterile 0.9% saline was injected through the triple-guarded catheter (MILA Triple Stage Catheter #EMAC800, MILA International, INC, Florence, KY, USA). The scope was advanced to the level of the horizontal trachea, and pooled fluid was immediately aspirated. Samples with at least 50% recovery were included. A 2.5 mL aliquot of sample was placed in a sterile tube for culture, and a second 2.5 mL aliquot was placed in an EDTA tube and submitted to the NCSU Veterinary Teaching Hospital Clinical Pathology Laboratory for a total nucleated cell count (TNCC) and cytology (performed by a masked board-certified clinical pathologist (EG)). The remaining fluid was placed on ice and processed within one hour.

An aliquot of the remaining fluid was saved for unfiltered direct measurement of cfDNA. The remaining TW fluid was filtered through gauze to remove excess mucus. Samples were centrifuged at 250× *g* for 10 min, and supernatant was harvested for analysis. Excess supernatant was stored at −80 °C for further analyses.

### 2.4. BALF Collection and Sample Processing

For the first group of horses (North Carolina), horses were sedated with detomidine (0.005–0.01 mg/kg IV) and butorphanol (0.02–0.04 mg/kg IV). For horses receiving both a BAL and TW, the TW was performed first endoscopically. Horses with severe asthma received intravenous Buscopan (0.3 mg/kg IV) prior to sampling for bronchodilation and bronchial smooth muscle relaxation. Topical lidocaine gel was applied to the nares. A cuffed catheter was passed nasotracheally until wedged in a secondary or tertiary bronchus (MILA Equine BAL Catheter 30Fr, 300cm #BAL300, MILA International, INC, Florence, KY, USA). The cuff was inflated with 4–6 mL of air, and 300 mL of sterile saline solution was infused and re-aspirated in two 150 mL aliquots. Only samples with at least 50% recovery were included. The aspirated fluid was pooled, submitted to NCSU Veterinary Teaching Hospital Clinical Pathology Laboratory, and a 500-cell count differential was performed by a masked clinical pathologist.

An aliquot of BALF was saved for unfiltered direct measurement of cfDNA. BALF was filtered through gauze to remove excess mucus. A TNCC was performed with Trypan blue and a hemocytometer. The samples were centrifuged at 250× *g* for 10 min. BALF supernatant was harvested, and an aliquot was stored at −80 °C for further analyses.

For the second group of horses (Université de Montréal), following sedation, diluted lidocaine (0.5%) was instilled into the airway and two boluses of 250 mL of warm isotonic saline were instilled into a main bronchus via a 1.6 m video endoscope. Fluid was aspirated with a suction pump and kept on ice until it was processed.

### 2.5. Cell-Free DNA Quantification

Cell-free DNA was measured in unfiltered/unspun BALF and TW and filtered/spun BALF and TW using the Qubit 4 fluorometer and 1X dsDNA HS assay kit (Thermo Fisher Scientific #Q33230, Waltham, MA, USA) per manufacturer protocols. Samples were measured in triplicate. BALF/TW supernatant cfDNA was also measured by spectrophotometry at 260 nm (NanoDrop 2000, Thermo Fisher Scientific) and SYTOX green plate-based fluorescence assay (Thermo Fisher Scientific #S34860) as previously reported [23,24]. For the SYTOX green plate-based assay, a standard curve was generated using serial dilutions of the 1X dsDNA HS high standard (10,000 ng/mL). DNA standards and samples were plated in triplicate, and SYTOX green nucleic acid stain was added to create a final well concentration of 1 µM. The plate was incubated at room temperature in the dark for 10 min prior to measuring fluorescence (excitation 485 nm, emission 528 nm) (Synergy HTX Multi-Mode Microplate Reader—Biotek, Winooski, VT, USA). Frozen supernatants were thawed, and cfDNA was measured with the Qubit 4 fluorometer and 1X dsDNA HS assay kit to determine if freeze/thawing affects cfDNA concentration. Absence of autofluorescence was confirmed through measurement of BALF supernatant samples without added SYTOX green/Qubit fluorescent working solution (Supplemental Table S2).

### 2.6. Data Analysis

Data were assessed for normality using the Shapiro–Wilk test. Parametric data were evaluated using a one-way analysis of variance (ANOVA) with multiple comparisons. Non-parametric data were evaluated using a Kruskal–Wallis test. Spearman correlation coefficients were calculated for correlation analyses. Analyses were performed using statistical software (GraphPad Prism 10.2.3), and significance was set at *p* ≤ 0.05.

## 3. Results

There were nineteen healthy horses with normal BALF cytology (<5% neutrophils, <1% eosinophils and <2% mast cells) and clinical respiratory scores < 5, eighteen horses with severe equine asthma (>20% neutrophils and clinical respiratory scores ≥ 15 at rest), fourteen horses with mild/moderate mastocytic equine asthma (>2% mast cells, <5% neutrophils and clinical respiratory scores 5–14) and twelve horses with mild/moderate neutrophilic asthma (6–20% neutrophils, <2% mast cells and clinical respiratory scores 5–14) (Supplemental Table S1) [25]. A subset of samples were used for correlation assays.

### 3.1. Qubit 4 Fluorometer Correlation

**Figure 1:**
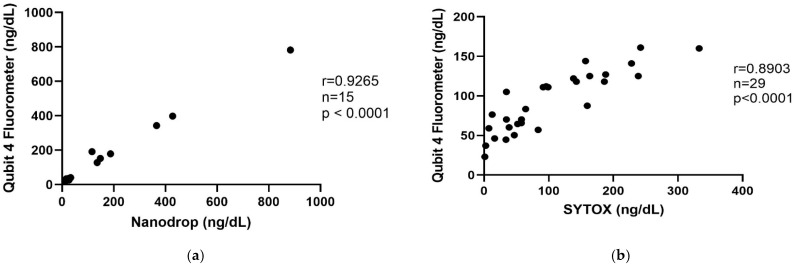
Cell-free DNA measured with the Qubit 4 fluorometer is strongly correlated with cell-free DNA measured with the NanoDrop (**a**) and SYTOX green fluorescence (**b**). (**a**) cfDNA was measured in BALF and TW supernatant with the 1X dsDNA HS assay kit and Qubit fluorometer and Nanodrop. (**b**) cfDNA was measured in BALF and TW supernatant with the 1X dsDNA HS assay kit and Qubit fluorometer and Biotek plate reader with SYTOX green. Spearman correlation.

There was a strong positive correlation (r = 0.9265, *p* < 0.0001, *n* = 15) between Qubit 4 fluorometer cfDNA measurement and NanoDrop cfDNA measurement in TW and BALF supernatant samples (Figure 1a). There was a strong positive correlation (r = 0.8903, *p* < 0.0001, *n* = 29) between Qubit4 cfDNA measurement and SYTOX green fluorescence plate-based cfDNA measurement in BALF and TW supernatant samples (Figure 1b).

### 3.2. cfDNA Measurement in Unfiltered vs. Supernatant

**Figure 2:**
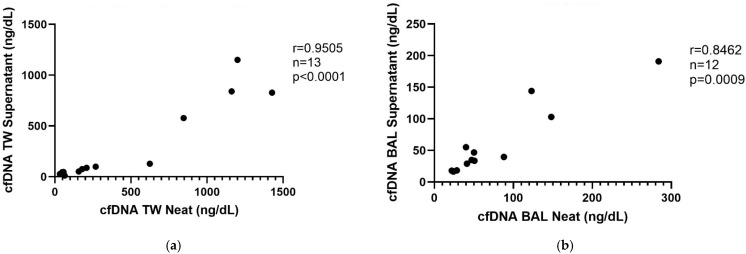
Cell-free DNA measured in TW supernatant (**a**) and BALF supernatant (**b**) is strong correlated with cell-free DNA measured in unfiltered TW (**a**) and unfiltered BALF (**b**). Cell-free DNA was measured with the 1X dsDNA HS assay kit and Qubit 4 fluorometer.

There was a strong positive correlation (r = 0.9505, *p* < 0.0001, *n* = 13) between cfDNA concentrations in unfiltered TW and TW supernatant with the Qubit 4 fluorometer and 1X dsDNA HS assay kit (Figure 2a). There was a strong positive correlation (r = 0.8462, *p* < 0.001, *n* = 12) between cfDNA concentrations in unfiltered BALF and BALF supernatant with the Qubit 4 fluorometer and 1X dsDNA HS assay kit (Figure 2b).

### 3.3. Frozen/Thawed Supernatant

There was a strong correlation (r = 0.9725, *p* < 0.0001, *n* = 20) between cfDNA concentrations measured in paired samples of BALF supernatant immediately following centrifugation and after one freeze (−80 °C)/thaw cycle. (Supplemental Figure S1).

### 3.4. TW vs. BALF cfDNA

There was no correlation between cfDNA concentrations in BALF supernatant and TW supernatant collected from the same horse (r = 0.1033, *p* = 0.7270, *n* = 14) (Supplemental Figure S2). There was a positive correlation between neutrophil percentage in BALF and TW collected from the same horse (r = 0.7657, *p* = 0.0021, *n* = 14) (Supplemental Figure S3).

### 3.5. cfDNA Quantification

**Figure 3:**
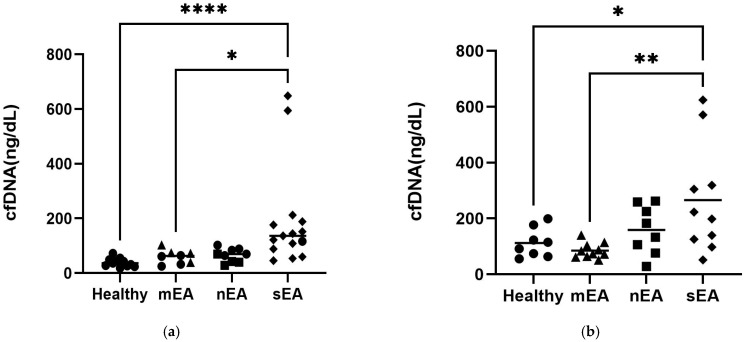
Cell-free DNA is significantly higher in BALF supernatant from horses with sEA compared to healthy horses and horses with mEA (mastocytic) in two groups of horses: client-owned horses/research horses in North Carolina (**a**) and horses from the Université de Montréal Equine Respiratory Tissue Biobank (**b**). (**a**) Healthy: *n* = 11, mild/moderate non-neutrophilic: *n* = 8, mild/moderate neutrophilic: *n* = 9, severe neutrophilic: *n* = 15. (**b**) Healthy: *n* = 7, mild/moderate non-neutrophilic: *n* = 10, mild/moderate neutrophilic: *n* = 8, severe neutrophilic: *n* = 10. Kruskal–Wallis. * = *p* < 0.05, ** = *p* < 0.01, **** = *p* < 0.0001.

There were two groups of horses analyzed. The first group consisted of NCSU-owned research/teaching horses and client-owned horses presenting with respiratory clinical signs. In this population, horses with sEA had significantly higher BALF supernatant cfDNA concentrations compared to healthy horses (*p* < 0.0001, *n* = 26) and horses with mild/moderate mastocytic equine asthma (*p* < 0.05, *n* = 23) (Figure 3a). The second population consisted of horses at the Université de Montréal Equine Hospital. Similarly, in this group, horses with sEA had significantly higher cfDNA concentrations in BALF supernatant compared to healthy horses (*p* < 0.05, *n* = 18) and horses with mild/moderate mastocytic equine asthma (*p* < 0.01, *n* = 20) (Figure 3b). In both populations, there was no difference in cfDNA concentrations between horses with mild/moderate neutrophilic equine asthma and the following groups: healthy horses, horses with mild/moderate mastocytic equine asthma and horses with severe equine asthma.

**Figure 4:**
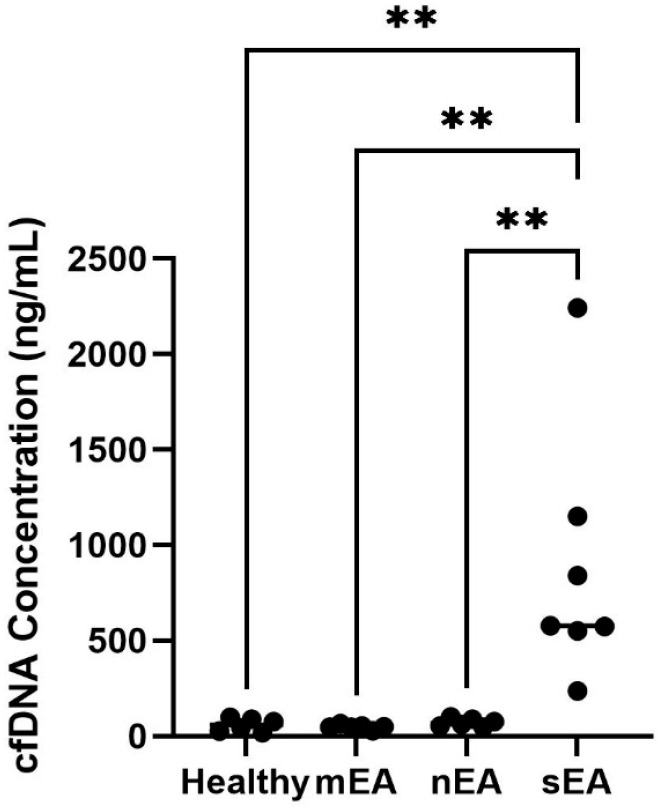
Cell-free DNA is significantly higher in TW supernatant from horses with sEA compared to healthy horses and horses with mEA (mastocytic and neutrophilic). Cell-free DNA was measured with the 1X dsDNA HS assay kit and Qubit 4 fluorometer. Population includes NCSU-owned research horses and client-owned horses in North Carolina. Healthy: *n* = 6, mild/moderate non-neutrophilic: *n* = 6, mild/moderate neutrophilic: *n* = 6, severe neutrophilic: *n* = 7. One-way ANOVA. ** = *p* < 0.01.

Tracheal wash samples were collected from the first group of horses in North Carolina. Horses with sEA had significantly higher cfDNA concentrations in TW supernatant compared to healthy horses (*p* < 0.01, *n* = 13) and horses with mild/moderate neutrophilic asthma (*p* < 0.01, *n* = 13) or mastocytic equine asthma (*p* < 0.01, *n* = 13) (Figure 4).

## 4. Discussion

This is the first study evaluating cfDNA concentrations in both BALF and TW fluid from healthy horses and horses with equine asthma. There were strong correlations of cfDNA concentrations measured using the Qubit 4 fluorometer and the NanoDrop spectrophotometer and the Qubit 4 fluorometer and a plate-based SYTOX assay, confirming the reliability of the Qubit 4 for cfDNA measurement in these sample types. Furthermore, our findings show that processing airway samples with filtration and centrifugation did not significantly alter cfDNA concentrations compared to direct measurements in unprocessed fluid. A single freezing–thaw cycle did not substantially impact cfDNA concentrations. This is relevant because it shows that processes that could potentially damage cells, such as centrifugation and freeze/thaw, did not affect cfDNA concentrations in this study.

The initial phase of the study focused on the methodology for cfDNA quantification. While it has been previously established that cfDNA can be measured in equine plasma (Qubit 4) and BALF (PicoGreen fluorescence plate-based assay), its quantification in tracheal fluid or using the Qubit 4 in respiratory samples has not been previously investigated [19,20,25]. SYTOX green, a cell-impermeant nucleic acid stain, only fluoresces when it binds to DNA that is extracellular or contained within a cell that has lost membrane integrity. This fluorescence can be measured quantitatively. The loss of cell membrane integrity and the release of extracellular DNA are characteristic features of NETosis. By combining the analysis from the NanoDrop and SYTOX green, we were able to confirm that the Qubit 4 fluorometer is an accurate, feasible method for cfDNA quantification in equine airway samples.

Previous studies suggest that human plasma can be stored at −80 °C for several years without affecting cfDNA concentration and that human plasma cfDNA concentrations do not differ significantly following three freeze–thaw cycles [26]. Our results agree and expand upon these findings; cfDNA was stable in lower airway samples and freezing supernatant (−80 °C) did not significantly change cfDNA concentration. This is the first study comparing measurement of cfDNA in unfiltered airway samples to cfDNA measurement in supernatant from filtered and centrifuged airway samples.

Interestingly, no correlation was found between cfDNA concentrations in paired BALF and TW supernatant samples. This is despite the fact that neutrophil percentages in these samples were positively correlated (Supplemental Figure S3). Positive correlation between TW and BAL neutrophils was also shown in a study by Rossi et al. [22]. Looking at our data in more detail, five of the horses with high concentrations of cfDNA in their TW had rather low levels of cfDNA in their BAL. However, the opposite was also true, with one horse having a high concentration of cfDNA in BALF but a low concentration of cfDNA in the TW (Supplemental Figure S2). Interestingly, all six of these horses had sEA. This could suggest different mechanisms and degrees of neutrophil activation at different levels of the airway in horses with severe asthma. For example, horses may experience different degrees of local tracheal irritation associated with tracheal mucus, which could promote neutrophil activation within the trachea. Secondary bacterial infection is another potential cause of variations in neutrophil activation, although none of the horses in this study had evidence of bacterial pneumonia. In the more distal airway, neutrophil activation is part of a cycle of inflammation triggered by organic dust. Another potential explanation for the lack of correlation between TW and BALF cfDNA concentrations is differing return volumes during lavage procedures, which could lead to variations in dilution factors for cfDNA concentrations. While we tried to minimize the impact of this variability (i.e., only samples with greater than 50% return were included in this study), there was a range in return volume (50–100%) Finally, this finding is likely impacted by the small sample size. It is worth noting that in our study, the TW samples had higher concentrations of cfDNA and greater discrimination between sEA and other groups. Additional research is needed to determine whether the sources of increased cfDNA in TW vs. BAL differ in horses with sEA.

Our findings are consistent with prior studies showing that cfDNA is higher in BALF supernatant of severe asthmatic horses as compared to healthy horses [25]. Across two geographically independent populations, horses diagnosed with severe equine asthma consistently had significantly higher cfDNA concentrations in BALF supernatant compared to healthy horses or those with mild/moderate mastocytic equine asthma. In contrast, no significant differences in BALF cfDNA were observed between horses with mild/moderate neutrophilic equine asthma and any of the other groups (healthy, mild/moderate mastocytic asthma or severe asthma). In the NC population, horses diagnosed with severe equine asthma had significantly higher cfDNA concentrations in TW supernatant compared to healthy horses, horses with mild/moderate neutrophilic asthma and horses with mild/moderate mastocytic asthma, supporting the use of cfDNA as a biomarker in this sample type. This finding is similar to human studies where cfDNA has been noted to be elevated in tracheal sputum in asthmatic patients [27].

This study had several limitations. First, not all of the horses’ samples could be included in the study due to a lack of adequate return volume (less than 50% of instilled volume), therefore not all horses had a paired BAL and TW. Further investigation into the lack of correlation between TW and BALF cfDNA is warranted. This could include examining potential correlation with mucus score, looking for subclinical bacterial infection, comparing NET markers in each sample type and using a urea assay to account for dilution. Additionally, it is important to note that there are other sources of cfDNA besides NETosis, including apoptosis and necrosis [28,29]. Therefore, we cannot be positive that NETosis is the only source of cfDNA in our samples. In future studies, we plan to investigate markers associated with other potential sources of cfDNA including apoptosis and necrosis. We also plan to quantify other components of NETs, such as citrullinated histone H3 or myeloperoxidase, along with cfDNA, in airway samples to determine whether NETosis is the primary mechanism of increased cfDNA in airways of horses with severe asthma.

## 5. Conclusions

In conclusion, this study supports the use of the portable Qubit 4 fluorometer and 1X dsDNA HS assay for cfDNA measurement in respiratory samples. Additionally, our findings support cfDNA as a potential biomarker of severe equine asthma. Given the ease of measurement, even in a field setting, further investigation of the clinical utility of cfDNA as a biomarker for respiratory disease is warranted. Additional research on the role of cfDNA in the pathophysiology of severe equine asthma is needed, as NETs remain a potential novel therapeutic target for severe equine asthma.

## Data Availability

The original contributions presented in the study are included in the article/Supplementary Materials; further inquiries can be directed to the corresponding author.

## Supplementary Materials

The following supporting information can be downloaded at https://www.mdpi.com/article/10.3390/ani15233483/s1, Table S1: Clinical scores and BALF cytology. Table S2: Autofluorescence of a subset of samples measured with SYTOX and the Qubit 4 fluorometer. Figure S1: Frozen/thawed BALF and TW supernatant cfDNA. Figure S2: Paired BALF and TW supernatant cfDNA. Figure S3: Paired BALF and TW neutrophil percentage.

## Abbreviations

The following abbreviations are used in this manuscript:

sEA	severe equine asthma
mEA	mild/moderate equine asthma
BALF	bronchoalveolar lavage fluid
TW	tracheal wash
NETs	neutrophil extracellular traps
cfDNA	cell-free DNA
