# Supplementary material for: A Portable Fluorometer Detects Significantly Elevated Cell-Free DNA in Tracheal Wash and Bronchoalveolar Lavage Fluid in Horses with Severe Asthma
Source: Animals (Basel). 2025 Dec 3;15(23):3483. doi: 10.3390/ani15233483 (PMC12691151; doi:10.3390/ani15233483)
Supplement: Supplementary file 1 [file animals-15-03483-s001.zip › animals-4000266-supplementary.pdf]

Table S1 | 23 point clinical score and cytology findings of horses enrolled in this study

		BALF Cytology (%)				
**Group**	**Clinical Score**	**Macrophages**	**Lymphocytes**	**Neutrophils**	**Eosinophils**	**Mast Cells**
Healthy	0	48	49.2	0.8	0.4	1.6
	0	54.6	39.4	3.8	0.6	1.6
	0	47.4	49.6	1.8	0	1.2
	1	61.8	35	1.8	0	1.4
	0	62	34.8	2.6	0	0.6
	0	72.5	23.9	2	0	1.6
	1	60.7	35	2.8	0	1.4
	2	62.7	32.9	3.7	0	0.7
	1	54.2	42.8	2.6	0	0.4
	3	61	35.2	3.2	0.2	0.4
	2	52.6	42	4	0	1.4
	0	38	59	2	0	1
	0	67	30	2	0	1
	0	50	46	4	0	0
	0	56.5	43	0.5	0	0
	0	59	37	3	0	1
	0	80.5	19	0	0	0.5
	0	34	60	4	0.2	1.8
	0	79	18	2	0	1
mEA (mastocytic)	0	66.6	27.4	3.4	0	2.6
	3	74.2	15.6	4	0.4	4.2
	2	78.2	17.4	1.4	0	3
	0	59	32.6	4.2	0	4.2
	1	55.4	36.2	3.2	0	5.2
	9	64.2	23.2	2.6	0.4	8.6
	4	59.4	30.2	4.6	0.6	5.2
	4	45	44	4	0	7
	6	52	40.2	4.8	0	3
	9	48	44.2	1	0.8	6
	4	47	50	0.8	0	2.2
	9	53	37	3	0	7
	4	44	45	1	0	10
	4	60	38	0	0	2
mEA (neutrophilic)	6	60.4	31.8	6.4	0.2	1.2
	2	62.8	18.8	19.8	0.2	0.4
	3	60.8	20.2	18.2	0.2	0.6
	5	55.6	24.2	18	0.4	1.8
	1	47.8	38	13	0.2	1
	4	73.4	17.6	8.2	0	0.8
	4	15	72	12.5	0	0.5
	4	53	33	12	1	1
	2	28	58	13	0	1
	2	42	42	14.5	0	1.5
	4	45	47	8	0	0
	2	37	45	18	0	0
sEA	15	38.2	29	30.8	0.4	1.6
	19	9.3	9.3	81.4	0	0
	15	5.4	13	81.2	0	0.4
	16	33	18.2	48.6	0	0.2
	18	38.5	36.625	24.25	0.5	1.125
	16	16.5	22.75	60	0	0.75
	16	26.375	17.375	55.5	0	0.75
	18	6	16.25	77.5	0	0.25

Supplementary Table S2. Autofluorescence of a subset of samples measured with SYTOX and the Qubit 4 fluorometer.				
	cfDNA Concentatrion			
**Group**	**Qubit**	**Qubit Control**	**SYTOX**	**SYTOX Control**
Healthy	24	0	29.14	0
	14	0	29.46	0
	50	0	54	0
	22	0	25.55	0.2
mEA (mastocytic)	56	0	58.7	0
	28	0	29.62	0
mEA (neutrophilic)	84	0	95.7	0
	18	0	10.6	0
	42	0	29.4	0
sEA (severe)	136	0	165.4	0
	88	0	85.85	0
	76	0	84.6	3
	264	0	249.4	1.5
	191	0	245.9	0

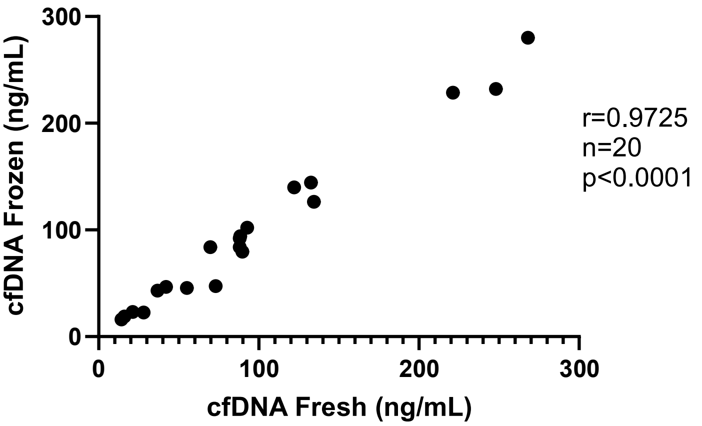

Supplemental Figure S1. Cell-free DNA measured in frozen/thawed BAL and TW supernatant is strongly correlated with cell-free DNA measured in fresh BAL and TW supernatant. Cell-free DNA was measured with the 1xdsDNA HS assay kit and Qubit 4 fluorometer. Spearman r correlation.

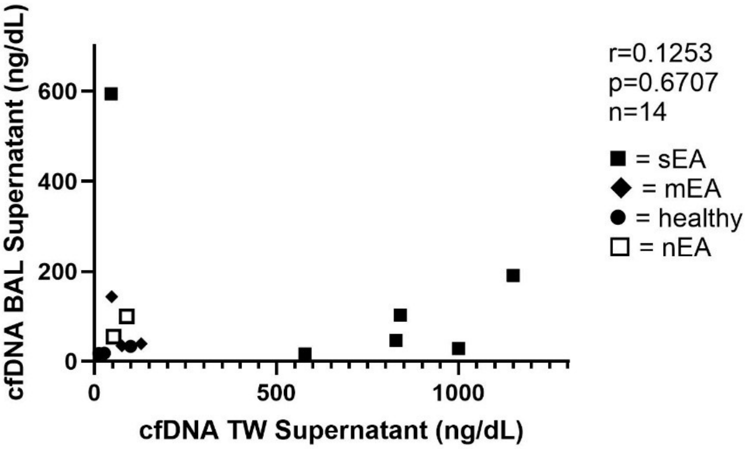

Supplemental Figure S2. Cell-free DNA measured in BAL and TW supernatants are not correlated. Cell-free DNA was measured with the 1xdsDNA HS assay kit and Qubit 4 fluorometer. Spearman r correlation.

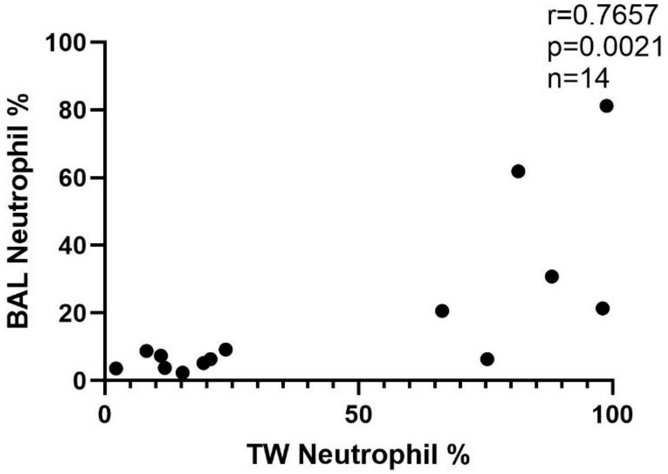

Supplemental Figure S3. Neutrophil percentages in TW and BALF are correlated. Spearman r correlation.
